# Relationality, Overload, and Trust: How Housing-Insecure Youth Navigated Health Information During the COVID-19 Pandemic

**DOI:** 10.3390/ijerph23070863

**Published:** 2026-06-30

**Authors:** Hannah E. Reynolds, Alana R. Lopez, Renatta Escobedo, Lorilee Chien, Samia Saeb, Jacob Carson, Jerel P. Calzo, Corinne McDaniels-Davidson, Steven Jellá, Jennifer K. Felner

**Affiliations:** 1 Institute for Behavioral and Community Health, San Diego State University Research Foundation, San Diego State University, 9245 Sky Park Court, San Diego, CA 92123, USA; alopez14@sdsu.edu (A.R.L.); jcalzo@sdsu.edu (J.P.C.); cmcdaniels@sdsu.edu (C.M.-D.); 2 School of Public Health, San Diego State University, 5500 Campanile Dr, San Diego, CA 92182, USA; ssaeb2552@sdsu.edu (S.S.); jcarson1279@sdsu.edu (J.C.); 3 Herbert Wertheim School of Public Health and Human Longevity Science, University of California, 9500 Gilman Dr. La Jolla, San Diego, CA 92093, USA; 4 San Diego Youth Services, 3255 Wing St, San Diego, CA 92110, USA

**Keywords:** COVID-19, health literacy, housing-insecure youth, social networks, trust

## Abstract

**Highlights:**

**Public health relevance—How does this work relate to a public health issue?**

- This study examines how youth experiencing homelessness and housing instability (YEH) navigated health information and guidance during the COVID-19 pandemic.
- These findings are relevant as current and emerging infectious disease threats present new challenges related to trust, misinformation, and access to public health guidance in the ‘post-pandemic’ era.

**Public health significance—Why is this work of significance to public health?**

- This study addresses a gap in research on health communication and health literacy among YEH during public health emergencies.
- The findings show how trust, relationships, and structural conditions shape engagement with health information and prevention guidance.

**Public health implications—What are the key implications or messages for practitioners, policy makers and/or researchers in public health?**

- Public health communication for YEH should prioritize trusted messengers, transparent information, and accessible communication strategies.
- Public health responses must address structural barriers, including housing instability and unmet basic needs, that limit youths’ ability to act on health guidance.

**Abstract:**

Youth experiencing homelessness and housing instability (YEH) face disproportionate health risks and structural barriers to health equity, which were intensified during the COVID-19 pandemic. We conducted a community-based participatory research study with 21 adolescents (ages 13–17) and transitional-aged youth (ages 18–26) experiencing homelessness or housing instability in San Diego, California, USA. Between February and July 2023, we conducted six “arts-based engagement sessions” (5 in English, 1 in Spanish) that blended expressive arts and focus group approaches to examine how YEH accessed, evaluated, and applied health information, and how trust and material and structural resource constraints shaped engagement with COVID-19 information and prevention guidance. We analyzed data via applied thematic analysis. Participants described how COVID-19 restrictions and economic disruption amplified pre-existing housing precarity, worsening material conditions and mental health. Participants selectively trusted COVID-19 information based on who provided it, how credible it felt, and whether it aligned with their lived experiences. Community-based providers and, for some, churches and cultural or ancestral knowledge were key information sources. Trust in government and public health messaging was conditional and shaped by perceptions of credibility, coherence, and political motivation. Participants described information overload and rapidly changing guidance as overwhelming and often incompatible with their material realities, leading to disengagement, reliance on intuition, or deprioritization of prevention behaviors. Effective communication during future health emergencies must center trusted relational messengers, align guidance with lived realities, and address the structural conditions that shape whether health information can be meaningfully acted upon.

## 1. Introduction

Homelessness and housing instability are associated with higher rates of morbidity and mortality across the life course [1]. In 2025, approximately 745,652 people in the U.S. were experiencing homelessness on a given night—a 27% increase since 2013 [2]. Homelessness is defined as lacking a fixed, regular, and adequate nighttime residence, while housing instability encompasses housing-related challenges, including difficulty paying rent, overcrowding, frequent moves, and reliance on temporary arrangements [2,3].

Youth experiencing homelessness and housing instability (YEH) include adolescents ages 12–17 and transitional-aged youth (TAY), typically ages 18–25. Youth bear a disproportionate burden of housing instability in the U.S.: more than one-quarter of people experiencing homelessness are under age 25, and nearly 3% of high school students report unstable housing [2,4]. Compared to stably housed youth, YEH evidence higher rates of depression, suicidality, substance use, and infectious and chronic diseases, alongside structural barriers to healthcare that increase the risk of long-term adverse health outcomes and premature mortality [4,5,6].

Although health disparities among housing insecure populations are well-documented, the COVID-19 pandemic renewed attention to the relationship between stable housing and health equity. Early in the pandemic, public health officials, social service providers, and researchers developed strategies to prevent COVID-19 transmission among people experiencing homelessness, emphasizing masking, social distancing, testing, and—once available—vaccination. The U.S. Centers for Disease Control and Prevention issued guidance to minimize COVID-19 risks among people experiencing homelessness [7], and local health departments across the U.S. implemented COVID-19 street outreach efforts [8]. However, these responses largely treated people experiencing homelessness as a homogenous population, with limited attention to the distinct developmental, social, and structural contexts shaping YEH’s experiences.

Health literacy—a three-tiered framework for the ability to access, understand, evaluate, and use health information to make health-related decisions—is particularly critical during public health emergencies [9,10]. Tailored health communication is essential for promoting health literacy and supporting decision-making among populations facing structural barriers and constrained opportunities to be healthy [11]. “Trusted messengers” play a central role in disseminating public health information, underscoring the importance of understanding where individuals access health information and how trust shapes its use [12].

For YEH, developmental stage, inequitable access to resources, and competing priorities related to basic needs may influence how health information is accessed, interpreted, and used [13]. Evidence among adults experiencing homelessness suggests higher health literacy is associated with better self-reported health [14]; tailored health education interventions improve vaccine attitudes [15]; and structural barriers and mistrust limit uptake of health guidance and healthcare disengagement [16]. Much less is known, however, about how YEH navigate health information during public health emergencies or how communication strategies can align with their lived realities, including the most effective trusted messengers for this population.

Limited attention to YEH during the COVID-19 pandemic represents a missed opportunity for health equity promotion—one that remains salient as public health prepares for future health threats. To address this gap, we used qualitative data to examine how YEH accessed, evaluated, and applied health information, and how trust and material and structural resource constraints shaped engagement with COVID-19 information and prevention guidance. Our study is situated in San Diego, California, a region with one of the highest rates of homelessness in the state [17].

## 2. Materials and Methods

This community-based participatory qualitative research study was driven by a community-identified priority and positioned a local community-based organization as an equitable research partner [18,19]. In 2021, researchers from a local university partnered with administrators and staff at a youth service organization to examine how YEH obtain and use COVID-19 prevention information, with the goal of informing policy and practice at the organization’s youth shelters and drop-in spaces.

### 2.1. Study Procedures

The study is guided by an exploratory qualitative approach in which we blended more traditional focus group methodology with arts-based research methods. We prioritized arts-based methods as a departure from the typical dynamics of health behavior research such that YEH could participate via various communication and expression styles [20]. Arts-based methods are characterized by the use of art in any form (e.g., fine arts, visual arts) to generate, interpret, and communicate knowledge and lived experiences, particularly in community-oriented research [21,22].

Drawing on the expertise of two expressive arts (“EXA”) therapist team members (R.E. and L.C.), we gathered data via “arts-based engagement (ABE) sessions” which blend traditional focus group methodology with EXA therapeutic traditions described above. This design intended to shift the dynamic of traditional unidirectional researcher–participant interaction to a bidirectional conversation that would increase participant engagement and produce more useful data to answer our key research questions [20]. We describe the ABE activities briefly below.

Eligible participants were ages 12–26, English- or Spanish-preferring, and had experienced homelessness, housing instability, or “couch-surfed” in the past six months [23]. Study team members from the youth service organization recruited participants from an emergency shelter for adolescents (the only overnight shelter for minors in San Diego at the time), a daytime drop-in center for youth, and through street outreach.

Between February and July 2023, we held six in-person ABE sessions with a total of *n* = 21 youth participants, including one session facilitated in Spanish. Each participant attended only one session. Based on guidance for qualitative data collection and application [24], three sessions were held with adolescents ages 13 to 17 years old (*n* = 9) and three sessions were held with TAY ages 18 to 24 years old (*n* = 12). Although youth ages 12 to 26 years old were eligible to participate, our sample only included those ages 13 to 24.

We planned *a priori* to conduct between 4 and 6 focus groups based on guidance for qualitative data collection [24], as well as the exploratory objectives and scope of the study. We aimed for representation from both adolescents and TAY, with the goal of collecting sufficient data to address the research questions and characterize major patterns across age groups. Although we originally planned to only gather data in English, we expanded data collection to include the perspectives of Spanish-preferring TAY based on recommendations from social service providers and interest in participation among Spanish-preferring TAY during recruitment. Sessions were conducted at an emergency shelter and daytime drop-in center and co-facilitated by EXA therapists (R.E. and L.C.) and research assistants (H.E.R., A.R.L., and J.C.). Sessions lasted on average 101 min (range: 80–123). See Table 1 for an overview of data collection.

Sessions included two activities related to participants’ experiences during the COVID-19 pandemic. In the first activity, participants drew, wrote, or collaged on “masks” (paper with string ear loops). One side represented internal feelings during the pandemic (e.g., emotions, thoughts, or experiences); the other side represented how they presented themselves externally during the pandemic (e.g., visible/outward emotions, social experiences). EXA therapists facilitated discussion about participants’ art and its meaning. In the second activity, participants responded to open-ended questions about experiences and feelings during the pandemic by selecting one or more provided laminated images (art, photographs, etc.) to represent their response (with the option to also write out their response). Research assistants facilitated group discussion about participants’ selection of cards and associated meaning. Questions included:

- How do your social circle and support networks (e.g., friends/peers, family, service providers) affect how you make decisions about your health?
- Where do you like to get information about health? ([if needed], for example, verbally from people you trust, social media, word of mouth, news, or somewhere else?)

Sessions were audio recorded. Facilitators took photos of participants’ artwork and wrote field notes during and after the sessions. The San Diego State University Institutional Review Board (IRB) approved this study. All participants provided informed assent or consent prior to participation. Participants aged 13–17 years provided written informed assent. The IRB approved a waiver of parental permission for these participants because requiring parental permission could have excluded youth with limited or no contact with parents or legal guardians, a circumstance common among youth accessing shelter and drop-in services. Furthermore, California law permits minors aged 12 years and older to independently consent to certain health and social services, including emergency shelter services. Participants aged 18 years and older provided verbal informed consent and were given the option to provide written informed consent. Participants received a $50 gift card for their time. Data were professionally transcribed verbatim, translated (Spanish to English), and de-identified.

### 2.2. Data Analysis

We leveraged an applied thematic analysis approach [25] using a 4-step process with a health equity lens [26]. Four analysts read and memoed transcripts and field notes [27] and drafted a codebook of inductive codes based on memos and deductive codes based on the questions/prompts. Analysts double-coded two transcripts and reconciled coding discrepancies [25] until achieving >90% agreement in code application [28]. All transcripts were then single-coded with the finalized codebook in NVivo (version 20.7.1) software. To develop themes, the lead author developed “code summaries” for selected codes, wrote analytic memos to capture key patterns in the data, and reviewed code reports to assess code saliency, repetition, and co-occurrence [26,29].

We refined and finalized themes through multiple rounds of team discussion and review. During theme development, we considered how participants’ experiences during the pandemic might relate to and diverge from stably housed youth to develop findings that would be most useful to providers serving this population. We triangulated findings across age groups and English and Spanish transcripts, examining convergence and divergence across transcripts and participants to identify differing perspectives [25,30].

### 2.3. Positionality & Reflexivity

We are investigators of diverse racial, ethnic, cultural, and social backgrounds at various career stages in social services and academia, including student trainees. We share some identities and social positions with the youth participants in the study, including some who were between 18–24 years old and some with lived experience of homelessness or housing instability. We engaged in group-level reflexive dialogue as we designed and conducted our study (e.g., weekly team meetings; data collection debriefs) and throughout data analysis (particularly between the first and last authors). Dialogue focused on methodological and ethical considerations, and reflections on our identities, social positions, and “insider” (social service provider) / “outsider” (academic) status vis-a-vis one another and our participants, and how these identities/positions shaped the study’s design, data collection processes, and data analysis [31,32].

## 3. Results

See Table 2 for sociodemographic information about participants who were on average 18.5 years old (range: 13–24). More than half reported a minoritized sexual orientation (57.1%) and/or race/ethnicity (88%), and all reported past 6-month housing instability. The demographic makeup of the sample aligns with prior research, which found that certain groups, particularly LGBTQ+ youth and youth of color, are overrepresented among YEH [33,34].

Our themes describe participants’ sources of health information during COVID-19 and the role of trust and material and structural constraints in accessing, evaluating, and using health information. We begin with a brief overview of participants’ housing-related experiences during early COVID-19 restrictions. Quotes in Spanish have been translated to English. See Table 3 for original quotes.

### 3.1. “Back Homeless”: COVID-19 and Housing Instability

Participants described connections between COVID-19 restrictions, economic disruption, and housing instability, including how the pandemic exacerbated instability:

*At the end of 2020, we got kicked out of the place we were living at so we were, actually, homeless for a good majority of COVID-19*. (Adolescent, S6, English)

*They fired everybody […] so I ended up getting kicked out of that rental assistance program. I was back homeless for a couple weeks*. (TAY, S2, English)

Overcrowding and safety concerns further shaped housing access and status among participants. Some participants described doubling up or living in cars or outdoors. One participant left stable housing because family members were not following COVID-19 precautions:

*I had family that were not being safe around others during COVID. It sometimes just felt like everyone around me was just not caring and was just being unsafe, and I felt just extra vulnerable. [So,] I couch-surfed a lot. Other times, I just hung out in parks*. (TAY, S1, English)

Despite experiences of housing loss and general instability, one participant described finding community within shelters:

*I was able to just be around people I didn’t have to fake for or put on a brave face for. I feel like I learned a lot. It was sad but I never really was scared*. (Adolescent, S1, English)

Overall, participants explained that COVID-19 amplified pre-existing housing precarity and worsened material and mental health conditions.

### 3.2. Primary Sources of Health Information: Trust and Relationality

Participants described several sources of COVID-19 health information: social networks, community institutions and service providers, and social media. Trust, perceptions of credibility, and strength of connection to information messengers shaped where and how participants sought information.

#### 3.2.1. “An Automatic Trust Thing”

Participants frequently identified family as trusted sources of information during the pandemic. In particular, adolescents emphasized parents and grandparents as primary sources of COVID-19 guidance, describing trust in family as instinctive:

*I would most likely go to my […] family [for information]. It’s just an automatic trust thing. It doesn’t matter how you feel against them. It’s just agreeing*. (Adolescent, S4, English)

*I would trust my mom or my grandparents for the news*. (Adolescent, S4, English)

Friends were also important sources of trusted information about COVID-19. For example, one participant explained how friends influence their masking behavior:

*When all of my friends didn’t have their masks on and stuff, I was like—because I was one of the only kids that did—I was like, ‘Hey, should I keep having this, or is everybody getting [COVID] anyways.’ They were like, ‘Yeah, you should just take it off and be yourself.’* (Adolescent, S5, English)

Further, concerns about misinformation and murky intentions reinforced reliance on friends and family One adolescent participant explained their reluctance to trust individuals outside of their immediate circle of friends and family:

*I can’t trust people [if they’re] not my family. Because what if you’re leading me down the wrong line? It’s like, who is who? You know? Everyone was masked up and you’re trying to find out, oh, who’s really my friend here?* (Adolescent, S4, English)

#### 3.2.2. Community Institutions and Providers

Participants described professionals within community institutions as key sources of information, including teachers, healthcare and social service providers, and for the group facilitated in Spanish, church leaders. One participant wrote that staff were their primary information source, as they, “push me to wear a mask and practice social safe distancing.” Trust in these providers was often rooted in ongoing relationships and consistent engagement.

Multiple participants in the group facilitated in Spanish shared that churches “were always talking about COVID,” and “giving recommendations,” about health and social resources. One participant noted some church messaging felt inaccurate, but they continued to attend because their mother sent them:

*My mom would send us to church. They were saying that the coronavirus wouldn’t hit the churches, and to keep going. I wouldn’t say anything*. (TAY, S3, Spanish [English translation], Quote 1)

Another participant emphasized the value of transparency, explaining that they would seek information from those in health-related fields or studying health:

*I would trust advice from certain people [who] I knew were working in that field for a long time […] It’s really important to know where things are coming from or who’s actually performing studies*. (TAY, S1, English)

#### 3.2.3. Culture and Ancestral Knowledge

Participants in the session facilitated in Spanish uniquely described the importance of cultural, religious, and ancestral knowledge when navigating the pandemic and seeking information:

*I use medications sometimes based on—with herbs […] from my ancestors […] natural remedies. Or witchcraft […] In some way, nature itself gives us the resources to be able to have good health*. (TAY, S3, Spanish [English translation]; Quote 2)

Multiple participants in this group shared that their local churches “were always talking about COVID” and “giving recommendations” about health and social resources. One participant, reflecting on negative experiences with the U.S. medical system, explained her preference for faith-based guidance:

*It is said faith moves mountains, so I prefer to trust in my faith and in God, and the gifts He’s given me […] So, the only thing left is to pray to God*. (TAY, S3, Spanish [English translation]; Quote 3)

#### 3.2.4. Social Media

Social media sites were common sources of COVID-19 information and guidance. Participants described high exposure to health updates online both via formal news outlets (e.g., CNN) and content shared by peers. For example, one adolescent participant explained the information they found on social media:

*Social media–I usually got updates about COVID and what was happening with the death percentages*. (Adolescent, S5, English)

Another participant explained that social media “influence[d] my behavior because everyone was on social media” (Adolescent, S6, English). Despite its ubiquity, participants described information accessed on social media as a less trusted source requiring skepticism and evaluation:

*I see all this information from […] social media […] and then I try to make my interpretation of it because it’s not always going to be true. So, you can also look it up and see if it’s true and make your own opinion on it*. (TAY, S1, English)

Information accessed via social media was heavily scrutinized by participants, and often weighed against the information learned from their immediate social circles.

Overall, participants relied on layered systems of trust when seeking COVID-19 information, favoring close relationships and lived experience and engaging institutional, digital, and cultural sources more cautiously.

### 3.3. “Make It Make Sense”: Conditional Trust in Government and the Role of Politics

Participants described government communications as important sources of information, but expressed concerns about trustworthiness, credibility, and utility of these sources. Some perceived information and policies as politically motivated:

*There was no logic behind anything that they were saying […] tell me how COVID-19 is not gonna come out after 10PM? Like, after that, COVID is out guys, like everybody has to shut down, you guys have to be in your house, but 9:59, like we’re good, we are still good, you don’t need a mask! Like…make it make sense*. (TAY, S2, English)

These evaluations directly shaped health behaviors for some participants, including vaccine uptake:

*[I didn’t get the vaccine because] I really just don’t trust the government. […] And then people were getting sick from it*. (Adolescent, S1, English)

Others described trusting government and public health institutions when information was perceived as evidence-based and responsive. One participant explained:

*Since we live under the government of the United States’ roof, […] I trust my life to them and the [Centers for Disease Control (CDC)]. […] I didn’t get COVID until the end of ’22. I followed all of the CDC rules, I was protected during the course of the pandemic, so I believe that following the science has its fruits*. (TAY, S3, Spanish [English translation]; Quote 4)

Another participant described watching television news, particularly CNN, with their family for up-to-date information regarding COVID-19, connecting this choice with their family’s political affiliation and the network’s use of statistics:

*We watched a lot of CNN. […] CNN had a lot of numbers every single day and they had that chart. […] The numbers at the bottom. The death rates. All of that. That’s why my grandpa really trusted CNN. Plus, we’re democrats […] that’s another reason why we watch CNN*. (Adolescent, S1, English)

Overall, participants described mixed feelings of trust and distrust in government information, driven by their evaluation of institutional intent, credibility, and coherence (“make it make sense!”).

### 3.4. Navigating Information Overload: Oversaturation and Incompatibility with Material and Structural Conditions

Participants described feeling overwhelmed by the constant influx of health information and misinformation from a variety of sources. Rapidly changing recommendations made it difficult to discern which sources and guidance to focus on and which prevention behaviors to adopt. For some, this led to avoiding COVID-19-related information:

*The news […] is just the worst thing for me because I’m already an overthinker. I just had to ignore it*. (TAY, S1, English)

*[T]here were so many different things being said […] I didn’t know what was true and what was false*. (Adolescent, S6, English)

For participants already experiencing stress due to housing precarity, constantly changing information exacerbated emotional and cognitive strain:

*[It] boosted my negatives and lowered my positives. It sucked out all my energy and [….] I felt like a caged animal. I felt like I couldn’t control some of my emotions because of the lockdown, and I was dealing with a lot. And so, it just really sucked out all of my energy on a day-to-day basis*. (TAY, S1, English)

Several participants acknowledged the importance of COVID-19 guidance, but described public health recommendations as competing with survival needs:

*You’re aware of the dangers [of overcrowding]. It’s just at some point you’d do anything to just sleep on a couch or something better than the floor I was staying*. (TAY, S1, English)

Some participants described relying on intuition or “instincts” when navigating overwhelming and contradictory information online:

*Participant A: There’s always new things on the internet, about everything, and a lot of ads on anything. So, all of the information is on there. […] You have your cell phone in your hands*.

*Moderator: How do you know which articles, videos, or online personalities are telling you things you can trust? How do you know who to trust and who not to trust?*

*Participant B: Because literally, we have a feeling, and we have to follow our instincts*.

*Participant A: Use your judgement—your intuition!*

(TAY, S3, Spanish [English translation]; Quote 5)

Overall, participants described oversaturation of information which led to disengagement or avoidance of COVID-19-related information and additional stress. For some, oversaturation led to relying on instincts or “vibes” to navigate information. Finally, public health guidance was often incompatible with participants’ material and structural conditions (e.g., social distancing in shelters) which directly shaped whether and how COVID-19 information was used.

## 4. Discussion

Our CBPR study examined how YEH accessed, interpreted, and applied health information during the COVID-19 pandemic. Participants described how the pandemic and associated restrictions exacerbated housing insecurity, disrupted social and economic stability, and contributed to emotional distress. Despite these challenges, participants generally reported an ability to access COVID-19 information and guidance from many sources, including friends, family, community-based providers, social media, and government agencies. However, our findings suggest that access and exposure to information alone did not determine whether it was trusted or used. Indeed, participants described trust as an important facilitator of information use, and suggested that applying COVID-19-related information and recommendations—even from trusted sources—was often shaped by their housing status or stability at the time. In other words, no matter how useful or trustworthy the guidance may have been, participants were often limited in their ability to apply it to support their health and well-being, given competing priorities and needs related to housing stability (e.g., social distancing is not possible or extremely difficult in congregate shelter settings). As a whole, these findings may suggest that access to information was necessary but largely insufficient for meaningful engagement with public health guidance among YEH.

Consistent with literature highlighting the importance of trusted messengers in public health communication [12], participants frequently identified family members, friends, and community-based service providers as highly trusted sources of information. Participants across groups discussed that trust was relational and often grounded in shared lived experiences and perceived credibility of the information source rather than institutional authority alone. Thus, participants described trust in government and public health institutions as conditional and largely dependent on their perception of the recommendations as evidence-based and sensible. One participant’s exasperated call to “make it make sense” explains this need for public health guidance to be scientifically backed, logical, and—perhaps most importantly—relevant to the contextual realities of homelessness and housing instability. For participants in the Spanish-language group in particular, cultural and religious information and practices heavily influenced trust in COVID-19-related information and behavior. Several participants in this group noted the relative importance of faith-based guidance compared to other sources of information. Taken together, these findings help explain previously documented distrust and limited uptake of COVID-19 information and public health recommendations among marginalized populations [35,36,37]. These results also further support evidence that trusted community organizations, including faith-based institutions, play a critical role in promoting engagement with health information and preventive health behaviors [12,38].

As mentioned previously, health literacy can be categorized into three levels: (1) functional health literacy, in which individuals possess basic skills to find health information and engage in preventive measures, (2) interactive health literacy, in which one can understand and apply new health information to changing situations, and (3) critical health literacy, enabling individuals to analyze diverse health information and exert control over health events [39]. Our findings suggest a relatively high level of health literacy among YEH in this study. For example, participants frequently described behaviors consistent with the “functional” and “interactive” levels of health literacy, including their processes of locating COVID-19-related information, comparing information across sources, evaluating the credibility of information, and questioning the validity of specific claims or guidance [39]. These findings nuance prior literature documenting low levels of health literacy among populations experiencing homelessness [40]. Rather, many participants described critically engaging with health information, particularly when information was shared through trusted social networks. Consistent with prior literature on health literacy, our participants’ emphasis on the important role of family members, friends, and community providers in their understanding and use of COVID-19-related information highlights health literacy as both an individual- and a community-level asset [39,41]. Participants described family members, friends, and community providers as playing active roles in their understanding of COVID-19-related information, suggesting that community-level health literacy shaped their individual-level health literacy and behavior. This aligns with calls to conceptualize health literacy as a multi-level asset and a modifiable social determinant of health [41]. From this perspective, health literacy is not only an individual skill but a resource that can be developed, reinforced, and enacted through social relationships and community contexts.

Notably, participants described a paradoxical relationship in which they had high engagement with COVID-19-related information on social media, but low trust in the information provided. This aligns with research suggesting social media is a primary source of news and health information for many young people [42]; however, our findings suggest that frequent exposure to information does not necessarily mean that YEH trust—and, in turn, use—information from social media. For example, participants described social media as a ubiquitous aspect of their daily lives that resulted in constant exposure to COVID-19-related content, but expressed skepticism toward the accuracy and credibility of information encountered online. Rather than functioning as a trusted messenger itself, participants described social media as a communication channel where they encountered and evaluated information and actively compared it across sources, often relying on trusted social ties to determine its validity. This distinction presents an important avenue for health communication efforts targeting YEH. For this population, social media’s value may lie in its ability to disseminate information broadly, while trust would need to be established through relationships with family, peers, and other trusted community members.

Importantly, even when participants trusted public health information and understood the recommended prevention behaviors (regardless of the source), material and structural realities constrained their ability to apply it. Participants described relying on shelters, couch surfing, and other crowded living arrangements where recommendations such as social distancing were difficult or impossible to implement. Others described prioritizing immediate survival needs, including securing housing and meeting basic needs, over adherence to prevention guidance. These findings align with health equity and socio-structural determinants of health frameworks [43,44], which emphasize that health behaviors are shaped not only by knowledge and motivation but also by access to resources and opportunities to be healthy [45]. In this context, housing instability functioned not merely as a backdrop to the pandemic but as a structural condition that shaped whether and how participants could engage with public health guidance. Together, these findings suggest that improving health literacy alone is unlikely to reduce health inequities among YEH without concurrent efforts to address the material conditions that constrain health-related decision-making.

### 4.1. Implications for Practice, Policy, and Research

The findings from this study can be instructive for service providers, policy makers, researchers, and other stakeholders who aim to promote health and well-being and mitigate health disparities among YEH. Our findings have implications for where, how, and by whom health-related information should be disseminated to YEH. We suggest that health-related guidance should be delivered by trusted messengers via social networks and online (e.g., social media) modalities in digestible quantities. The source should be clearly identified (e.g., the CDC) and linked to supporting scientific evidence to ensure transparency and trustworthiness. Even when supported by scientific evidence, institutions must function in ways that earn public trust, including among YEH. As our findings suggest, when information is not clearly linked to scientific facts or logic (e.g., 10 p.m. curfews during COVID-19 lockdowns), trust is threatened. Notably, health promotion interventions for YEH should not rely on information provision alone to influence behavior change. Evidence suggests that improving health literacy alone does not necessarily lead to healthy behavior (e.g., vaccine uptake) or address structural barriers to health-promoting resources [46].

Our findings suggest that public health communication efforts targeting YEH should leverage the ubiquity and reach of social media while pairing messages with trusted messengers and community-based organizations that can enhance credibility and promote engagement.

#### 4.1.1. Service Providers & Social Service Settings

Service providers and youth-focused professionals in youth service and educational settings are among the most important trusted messengers with the power to help YEH overcome material and structural barriers to identifying, evaluating, and using health information to support their well-being.

These professionals can work with YEH individually to share information, support evaluation, and—perhaps most vitally—link youth to health resources. For example, service providers within youth shelters and drop-in centers can work with youth via one-on-one case management to disseminate needed information, help youth evaluate the trustworthiness and utility of the information, and support youth in using the information to make health-related decisions (e.g., vaccine uptake) and access health resources and supports accustomed to serving YEH (e.g., local federally qualified health centers with youth clinics or programs). In addition, professionals in these settings can collaborate with local public health professionals to translate and disseminate information at multiple organizational levels to improve health literacy and shape health-related decision-making among YEH, including via organizational policies, staff training and supports, and group-level interventions for youth.

#### 4.1.2. Public Health Practitioners & Policymakers

Public health practitioners and policymakers should avoid one-size-fits-all communication strategies when developing and disseminating guidance for YEH. Our findings suggest that guidance is more likely to be engaged with when it is clearly linked to evidence, makes sense in the context of YEH’s lived realities, and is delivered by trusted messengers. Public health agencies should not assume that credibility is conferred solely by institutional authority; instead, they should prioritize transparency, coherence, and youth-informed approaches that acknowledge the material constraints experienced by YEH. Policies and guidance misaligned with YEH’s conditions (e.g., social distancing in shelters) risk undermining trust and limiting uptake.

#### 4.1.3. Researchers

Future research should further examine health literacy among YEH as a modifiable social determinant of health [41]. Additional work is needed to understand how health literacy develops within social networks, how trusted relationships shape information evaluation and decision-making, and how health literacy is partially predicated by access to material resources. Researchers should also evaluate interventions that both strengthen health literacy and address youths’ ability to act on health information. Given the increasing role of social media in health communication and the continued threat of infectious disease outbreaks and health-related mis- and disinformation, identifying effective strategies to support health literacy development and salubrious health behaviors among YEH remains a public health priority.

### 4.2. Strengths and Limitations

Our findings should be interpreted considering our study’s strengths and limitations. Data were collected between February and July 2023, approximately three years after the initial lockdown; although all participants experienced homelessness or housing instability at the time of data collection, they were not necessarily unhoused throughout the entire pandemic. This time gap may have influenced responses but also allowed participants to reflect across multiple stages of COVID-19 and compare their pandemic-era behaviors against their current behaviors. A key strength is our use of CBPR-informed, arts-based methods, which may have enhanced youth engagement and expression. Sampling from a single community-based organization in San Diego may limit transferability, though findings may be relevant to other urban contexts with high rates of youth homelessness. Further, sampling directly from a youth service organization may bias answers to reflect a preference towards information gathered from service providers. However, this organization is the largest of its kind and the sole overnight shelter for teens in the county. Finally, only one session was conducted in Spanish; future research with YEH should prioritize multilingual data collection.

An important strength of the study is that participants in our sample were diverse in their sexual orientation, gender identity, and race/ethnicity—reflecting the over-representation of LGBTQ+ youth of color (particularly Black and Latinx youth) among YEH in San Diego [47]. We were limited in our ability to examine experiences by participants’ individual and intersecting identities and social positions (e.g., race, gender identity, housing history); however, we suggest that because of the diversity of our sample, the findings should be understood within the context of the macro-level systems of oppression (i.e., structural racism, heterosexism, cisgenderism) that shape access to resources and opportunities among youth and adults experiencing homelessness in the U.S., particularly those who have multiple, intersecting minoritized identities and social positions [48,49].

## 5. Conclusions

The purpose of this qualitative CBPR study was to examine how YEH navigated health information during the COVID-19 pandemic. Findings suggest that COVID-19 restrictions and housing instability were closely intertwined and shaped participants’ health, housing experiences, and engagement with public health guidance. Although participants generally demonstrated substantial health literacy and were able to access and interpret COVID-19 information, whether guidance was applied depended on trust in the source and was often constrained by material conditions. These findings point to the need for public health approaches that better account for the lived realities of YEH when communicating urgent health information. While focused on COVID-19, this study has broader implications for health communication with YEH in what is considered the ‘post-COVID’ era, particularly as the U.S. and the world navigate emerging disease threats.

## Figures and Tables

**Table 1:** ABE sessions by age, language, sample size, and duration (*n* = 21).

Session	Age Range	Language	*n*	Session Duration (mm:ss)
1	18–24	English	5	101:54
2	18–24	English	2	101:29
3	18–24	Spanish	6	123:27
4	13–17	English	4	80:34
5	13–17	English	2	110:51
6	13–17	English	2	92:12

**Table 2:** Arts-Based Engagement Session Participant Demographics (*n* = 21).

Characteristics	*n*	Percentage (%)
Age		
Adolescents (13–17)	9	42.9%
Transitional Age Youth (18–24)	12	57.1%
Race/Ethnicity *		
Hispanic/Latinx	12	57.1%
African American/Black	5	23.8%
American Indian/Alaskan Native	5	23.8%
White	1	4.8%
Other	1	4.8%
Missing	1	4.8%
Gender		
Male	6	28.6%
Female	11	52.4%
Transgender	2	9.5%
Genderqueer	1	4.8%
Missing	1	4.8%
Sexuality		
Heterosexual	8	38.1%
Gay/Lesbian	4	19.0%
Pansexual	1	4.8%
Bisexual	7	33.3%
Missing	1	4.8%
Past-6 Month Housing Status *		
Sharing Housing	6	28.6%
Emergency/Transitional Housing	12	57.1%
Motels, hotels, trailer parks, campgrounds	4	19.0%
Areas not designed for human sleeping accommodation	1	4.8%
Cars, parks, public spaces, substandard housing, abandoned places, transit stations	1	4.8%
Missing	2	9.5%
Highest Education Completed		
Less than a high school graduate	9	42.9%
High school/GED	8	38.1%
Some college	3	14.3%
Missing	1	4.8%

* Not mutually exclusive; may not sum to 100 percent.

**Table 3:** English translations of verbatim Spanish quotes.

Quote 1	
*My mom would send us to church. They were saying that the coronavirus wouldn’t hit the churches, and to keep going. I wouldn’t say anything.* (TAY, S3, Spanish [English translation])	*Mi mamá nos mandaba para la iglesia. Pues decían que en las iglesias no pegaba el coronavirus, y que ándele, llegaran. Yo no decía nada.* (Spanish original/verbatim)
Quote 2	
*I use medications sometimes based on—with herbs […] from my ancestors […] natural remedies. Or witchcraft […] In some way, nature itself gives us the resources to be able to have good health.* (TAY, S3, Spanish [English translation])	*Yo utilizo medicamentos a veces a base de—con hierbas a través de […] De mis antepasados […] Naturales. O brujería. De alguna manera, la naturaleza misma nos da los recursos para poder tener buena salud*. (Spanish original/verbatim)
Quote 3	
*It is said faith moves mountains, so I prefer to trust in my faith and in God, and the gifts He’s given me […] So, the only thing left is to pray to God*. (TAY, S3, Spanish [English translation])	*La fe, dice que mueve montañas, así que prefiero confiar en mi fe y en Dios, y los dones que me ha dado. […] Entonces, lo único que quedaba era pedir a Dios*. (Spanish original/verbatim)
Quote 4	
*Since we live under the government of the United States’ roof, […] I trust my life to them and the CDC. […] I didn’t get COVID until the end of ’22. I followed all of the CDC norms, I was protected during the course of the pandemic, so I believe that following the science is like it has its fruits*. (TAY, S3, Spanish [English translation]	*Pues, como vivimos bajo el techo del gobierno de Estados Unidos […] Así que, les confío mi vida a ellos, y a la CDC. Porque siempre está como, cambiando y siempre la están como, actualizando. [N]no me dio COVID hasta el ’22, como al final. Seguí todas las normas de la CDC, estuve protegida por el transcurso de la pandemia, so yo creo que seguir la ciencia es como de que tiene sus frutos*. (Spanish original/verbatim)
Quote 5	
*Participant A: There’s always new things on the internet, about everything, and a lot of ads on anything. So, all of the information is on there. […] You have your cell phone in your hands.* *Moderator: How do you know—similar to what we were talking about with doctors—how do you know when… the internet has lots of different opinions and information that sometimes contradict each other? How do you know which articles, videos, or online personalities are telling you things you can trust? How do you know who to trust and who not to trust?* *Participant B: Because literally, we have a feeling, and we have to follow our instincts.* *Participant A: Use your judgement—your intuition!* (TAY, S3, Spanish [English translation])	*Participant A: En internet siempre salen muchas cosas, de todo, y un montón de anuncios de cualquier cosa. Así que, acá está toda la información. […] el celular lo tienes en la mano.* *Moderator: Cómo sabes—similar a lo del doctor, cómo sabes cuando—el internet tiene muchas diferentes opiniones y mucha información que a veces se contradice. ¿Cómo sabes qué artículos o videos, o personajes del internet que te están contando cosas, cómo sabes con quién confiar y con quién no confiar?* *Participant B: Porque literalmente, uno tiene un presentimiento, y hay que seguir a los instintos uno.* *Participant A: Por criterio, no. Vibra alto*! (Spanish original/verbatim)

## Data Availability

The data generated and analyzed during this study are not publicly available due to the sensitive nature of the qualitative data and the risk of participant identification.
